# Insight into the relationship between type IV pilus function and biofilm formation

**DOI:** 10.1042/BST20250151

**Published:** 2026-06-03

**Authors:** Yafan Yu, Robert M. Bauer, Rabab Mahdi, Kurt H. Piepenbrink

**Affiliations:** 1 Department of Biochemistry, University of Nebraska-Lincoln, Lincoln, NE 68588, U.S.A.; 2 Department of Food Science and Technology, University of Nebraska-Lincoln, Lincoln, NE 68588, U.S.A.; 3 Department of Chemistry, University of Nebraska-Lincoln, Lincoln, NE 68588, U.S.A.; 4 Nebraska Food for Health Center, University of Nebraska-Lincoln, Lincoln, NE 68588, U.S.A.; 5 Center for Integrated Biomolecular Communication, University of Nebraska-Lincoln, Lincoln, NE 68588, U.S.A.

**Keywords:** antigens, Biofilm, host-microbe Interactions, Protein DNA interactions, Type IV pili

## Abstract

Type IV pili (T4P) are protein nanofibers that can be extended and retracted from the surfaces of many bacterial taxa. They are involved in many aspects of bacterial physiology that differ between bacterial species, including surface motility, DNA uptake, and host-cell adherence, but genetically and structurally distinct type IV pilus systems from distantly related bacterial species have also been found to promote the formation of bacterial biofilms. The molecular mechanisms underpinning the promotion of biofilm remain an area of active investigation and may be both manifold and variable between type IV pilus systems. Two areas of recent interest are interactions between T4P and extracellular DNA and the relationship between surface-adhered biofilms and suspended aggregates. In the present review, we critically discuss the current state of knowledge of type IV pilus function and how these structures may interact with other biomolecules to influence the formation of multicellular bacterial communities. We examine the evidence for how alterations in DNA-binding, pilus retraction, and pilus composition have downstream effects on the formation of bacterial biofilms.

## Introduction: type IV pili as intercellular connectors

Type IV pili (T4P) are the best-studied members of a broader class of extracellular fibers collectively referred to as type IV filaments [1–3], which also includes competence pili [4] and tad/flp pili [5]. The basic structure of these fibers, which is also shared by type II secretion system (T2SS) endopili [6–8] and the archaellum [9,10], is a helical assembly formed from subunits extracted from the membrane. These subunits, pilins, all contain an N-terminal secretion tag (proteolytically removed before extraction by a prepilin peptidase) followed by a single transmembrane alpha helix at their N-terminus and terminate in a highly variable soluble C-terminal domain [4,5,11]. Although all known T4P systems encode multiple pilin subunits, a single subunit generally predominates (commonly referred to as the major pilin). However, some T4P systems contain multiple high-abundance pilins, which form structurally distinct fibers [12], and others include multiple subunits at high abundance within a single heteropolymer [13].

Historically, T4P were strongly associated with adhesion to extracellular DNA (eDNA) in natural competence, the process of bacteria acquiring novel genetic information from their environment by taking up DNA and incorporating those genes into their own genomes [3,14–17]. The seminal experiment demonstrating competence by Fredrick Griffith relied on DNA uptake through competence pili by *Streptococcus pneumoniae* [18]. This function was found to be conserved in *Neisseria gonorrhoeae* (and *Neisseria meningitidis*) [19], *Vibrio cholerae* [20], *Acinetobacter baylyi* [21], *Haemophilus influenzae* [22], and many other diderm (Gram-negative) bacterial species through T4P. Fundamentally, DNA uptake requires three molecular mechanisms: (i) extension of the pilus fiber, (ii) adhesion to DNA, and (iii) retraction of the pilus fiber through a reverse process of depolymerization. While gaps in our understanding of the molecular basis for DNA uptake remain, extensive progress has been made in identifying the T4P subunits that adhere to DNA [23–27].

Twitching motility, a type of surface motility that can function even at the interface of a solid and semi-solid medium [28,29], is also inherently dependent upon adhesion. T4P are extended, adhere to semisolid surfaces, and, upon being retracted, pull the bacterium forward as they are shortened. This makes twitching motility, like DNA uptake, totally dependent upon the ability of bacterial cells to depolymerize and retract T4P, but surface adhesion is also a prerequisite, making motility dependent upon the ambient salt concentration [30] as well as the elasticity of the medium [31].

Other adhesive interactions by T4P have generally been found to be independent of retraction and, in some cases, are enhanced when retraction is inhibited [32,33]. Adhesion by T4P pili is multifactorial, and specific adhesive subunits can be incorporated into tip complexes [34] or along the length of the pilus fiber [11,35,36]. These subunits are more variable in terms of their number, structure, and specific activity than the major subunits [37,38], which have generally been found to lack specific adhesive activity [23,25].

Here, we examine the current state of the field in terms of our knowledge of the molecular mechanisms by which T4P promotes the formation of bacterial biofilms, microcolonies, and other aggregates. In particular, we compare the known ability of various T4P systems to mediate the attachment of surface biofilms and bacterial self-association and discuss the potential for anti-T4P compounds or biologics as potential therapeutics for bacterial infections.

## Type IV pilus structure

**Figure 1:**
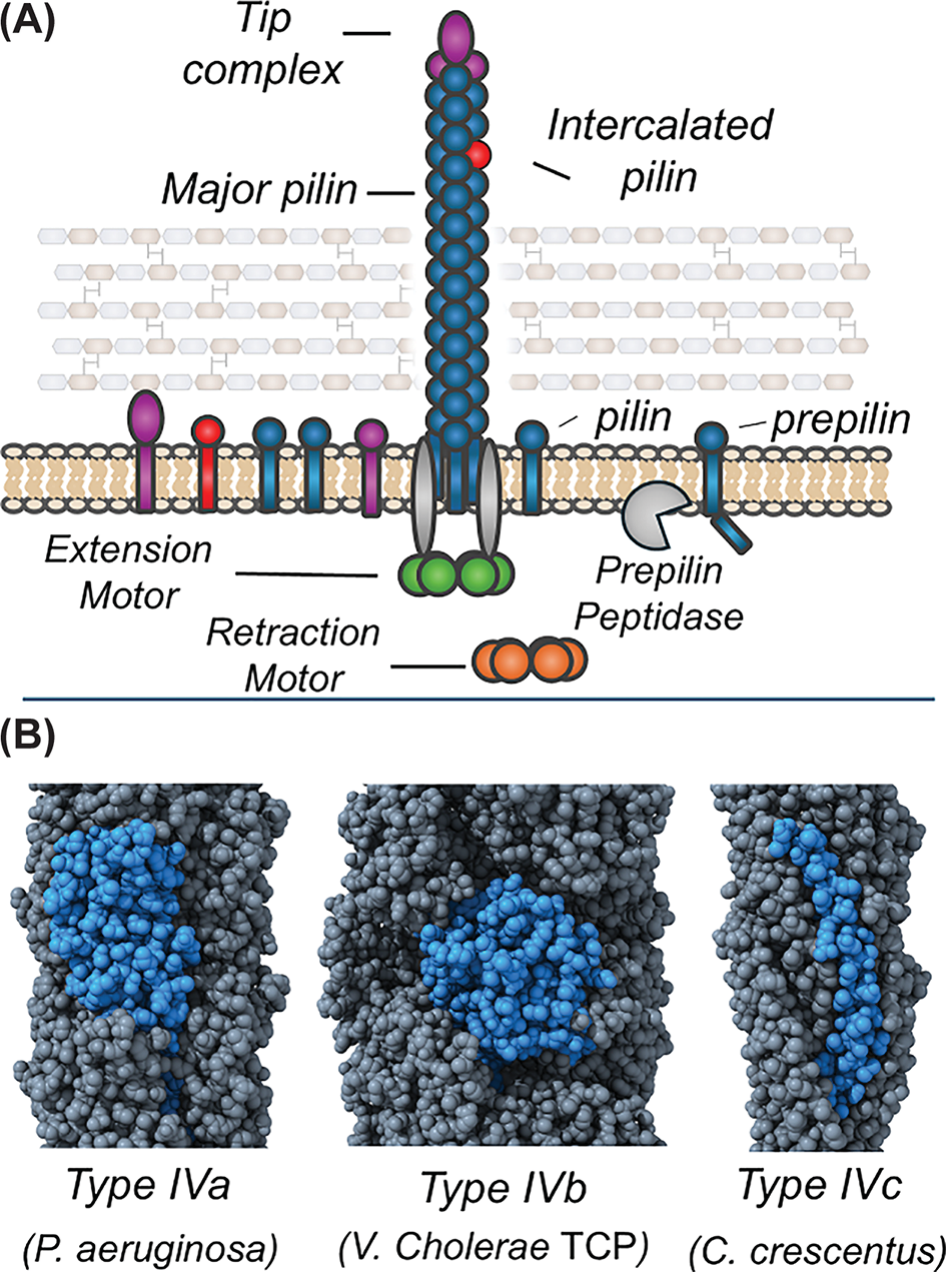
Type IV pilus fibers (**A**) Schematic of pilus biogenesis showing the pilins of the tip complex (purple), the major pilin (blue), intercalated pilins (red), and motor proteins PilB (green) and PilT/U (orange). The prepilin peptidase is shown in gray, processing the prepilin form of the major piilin. (**B**) Structures of pilus fibers from *Pseudomonas aeruginosa* (8TUM), the toxin-coregulated pilus (TCP) of *V. cholerae* (8UHF), and the *Caulobacter crescentus* (8U1K) tad pilus. Pilus fiber structures are depicted using ChimeraX [155]. To clarify the subunit boundaries, one subunit in each fiber is in blue rather than gray.

T4P are extended from the membrane (the inner membrane of diderms and the plasma membrane of monoderms) through the action of a complex machine spanning the membrane with hexameric AAA+ ATPase motors in the cytosol rotating to extract pilin subunits from the membrane and incorporate them into the growing fiber. These pilin subunits (and their counterparts in com pili and T2SS endopili) are universally proteolytically processed by a prepilin peptidase to remove N-terminal signal sequences to allow for extraction, depicted in Figure 1 as the transition from prepilin to pilin. Because new subunits are added to the base of the fiber, the initiation of pilus biogenesis appears to be preceded by the formation of complexes of specific subunits found at the pilus tip (see Figure 1A). These tip complexes, analogous to those found in T2SS complexes [39], frequently contain large subunits that could not be incorporated along the length of the fiber and are highly variable, including the incorporation of novel domains [27,40,41].

**Figure 2:**
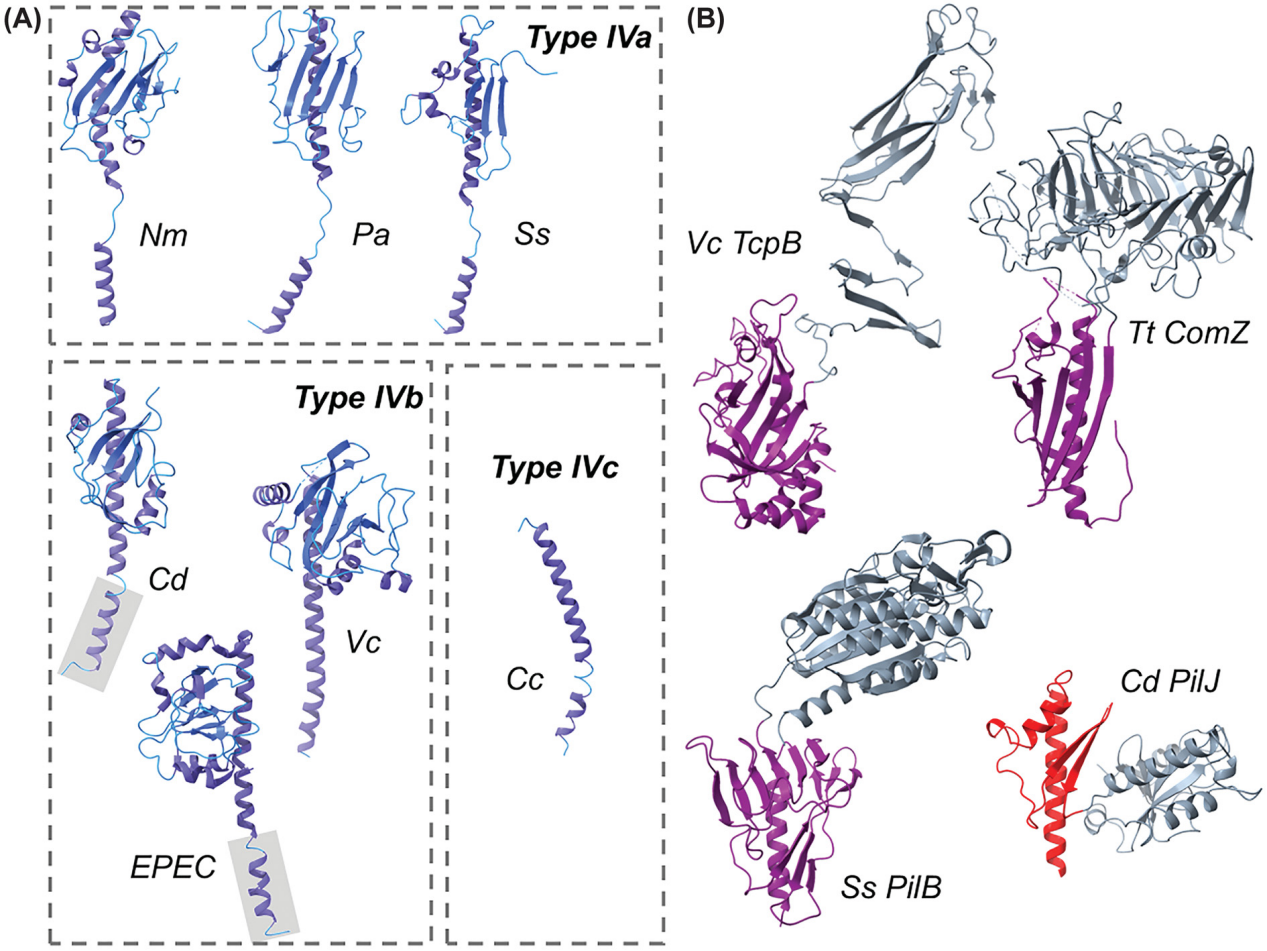
Structural variation in T4P subunits (**A**) Major pilin structures from type IVa, type IVb, and type IVc (tad/flp) pilus fibers. The N-terminal TM-helices of Cd PilA1 and enteropathogenic *E. coli* (EPEC) BfpA are modeled using AlphaFold 3 [156]. (**B**) Structures of pilin proteins with insertion domains: insertion domains are colored gray with the pilin domain colored purple (for those predicted to be at the pilus tip) or red (for PilJ, intercalated throughout the pilus fiber).

Pilin subunits are unified in their basic structure with an N-terminal signal peptide followed by a transmembrane α-helix (α1-N) followed by a soluble region beginning with a soluble α-helix (α1-C) and generally followed by a flexible loop (the αβ-loop) and a β-sheet packed against the α1-C helix. While transmembrane α-helices from any source (including the α1-N helices of pilin proteins) strongly resemble each other, the C-terminal soluble region, commonly referred to as the pilin head group, is responsible for the variation in the structure of pilin subunits. As described below, type IVb pilins are somewhat larger than their type IVa counterparts, and the type IVc pilin headgroups consist of only an α1-C helix. Although all known T4P systems encode multiple proteins predicted to be pilins based on their sequences, generally only one, or occasionally two [13], of those subunits is found to be highly expressed [42,43]. These subunits (commonly referred to as major pilin subunits) vary in size (Figure 1B), particularly in the incorporation of additional α-helices at the C-terminus (Figure 2A), found in the type IVb pilins of diderm (Gram-negative) bacteria [44] as well as PilA1 from *Clostridioides difficile* [45]. In Figure 2, we include PilE1 of *Streptococcus sanguinis* and PilA1 of *C. difficile* in the type IVa and type IVb categories, respectively, based on their structural similarities to proteins from those categories.

Some T4P systems have also been proposed to form pilus fibers using N-terminal fragments terminated somewhere in the αβ-loop. In *N. gonorrhoeae* strains with class I T4P systems, complementation of a *pilE* (the major pilin subunit) mutant with a truncated gene encoding for an N-terminal fragment is sufficient to restore DNA uptake but not other T4P functions [46], suggesting that the N-terminal fragment may form some structure on its own. In *Geobacter sulfredens*, the N- and C-terminal pilin sequences are expressed as separate gene products [47], but the N-terminal fragment has also been proposed to form fibers without the C-terminal gene product [48]. Tad/flp pilus systems (Type IVc, see Figure 2B) also encode a short, completely helical pilin gene product, and these truncated subunits form pilus fibers without the addition of an additional protein [49]. The α1-N helix is invariably ∼25 residues to span the length of a lipid bilayer; the α1-C helices described here vary somewhat in length. The *Geobacter* N-pilin protein is 59 residues, *C. crescentus* PilA is 45, and as few as the N-terminal 38 residues of *N. gonorrhoeae* maintain competence.

In some T4P systems, the pilus fiber is decorated with other subunits at low abundance. These subunits are incorporated at such low abundance that they offer little advantage for the stability of the pilus fiber, but these additions allow for the incorporation of adhesive moieties into the pilus fiber. To distinguish these subunits from those found at the tip, we refer to them here as intercalated pilins. PilJ, in *C. difficile*, was the first of these intercalated pilins to be discovered [11,45], but this same pattern of incorporation was found for PilV in *N. meningitidis* [35] and proposed for *C. difficile* PilW [25].

All T4P systems also appear to be extended only after the formation of an initiation complex, which consists primarily of specialized pilin subunits but can also include non-pilin proteins (PilY in *Pseudomonas/Acinetobacter*, PilC in *Neisseria*) [34,50]. These complexes are extended outward by pilus extension, residing at the pilus tip, and are highly variable. But the general architecture appears to be conserved between not only multiple T4P systems but also competence pili and T2SS. The tip complex of GspI, GspJ, and GspK from the *Escherichia coli* T2SS system provides the best insights available to us in terms of the structures of the analogous T4P tip complexes [39]. The spatial constraints of the tip are relaxed when compared with the pilus fiber, and several pilins from these initiation complexes contain large insertion domains [27,40,41,51] (Figure 2B), in some cases larger than the pilin domain. Because these additional domains are not constrained by the repeating structure of the pilus, they represent an obvious mechanism for the incorporation of adhesive activity into T4P (e.g., *T. thermophilus* ComZ has a β-solenoid insertion domain) [27].

## T4P retraction in bacterial biofilms

T4P are involved in a myriad of physiological processes, all of which are based fundamentally on the combination of two or three common mechanisms: pilus extension, adhesion (broadly defined), and pilus retraction. The promotion of biofilm formation by T4P likely involves multiple mechanisms, and several pieces of data, detailed below, suggest that incorporation into biofilms impacts T4P function, potentially through altered T4P expression, differential expression of complementary gene products, or the spatial constraints of the biofilm environment.

### Surface motility

**Figure 3:**
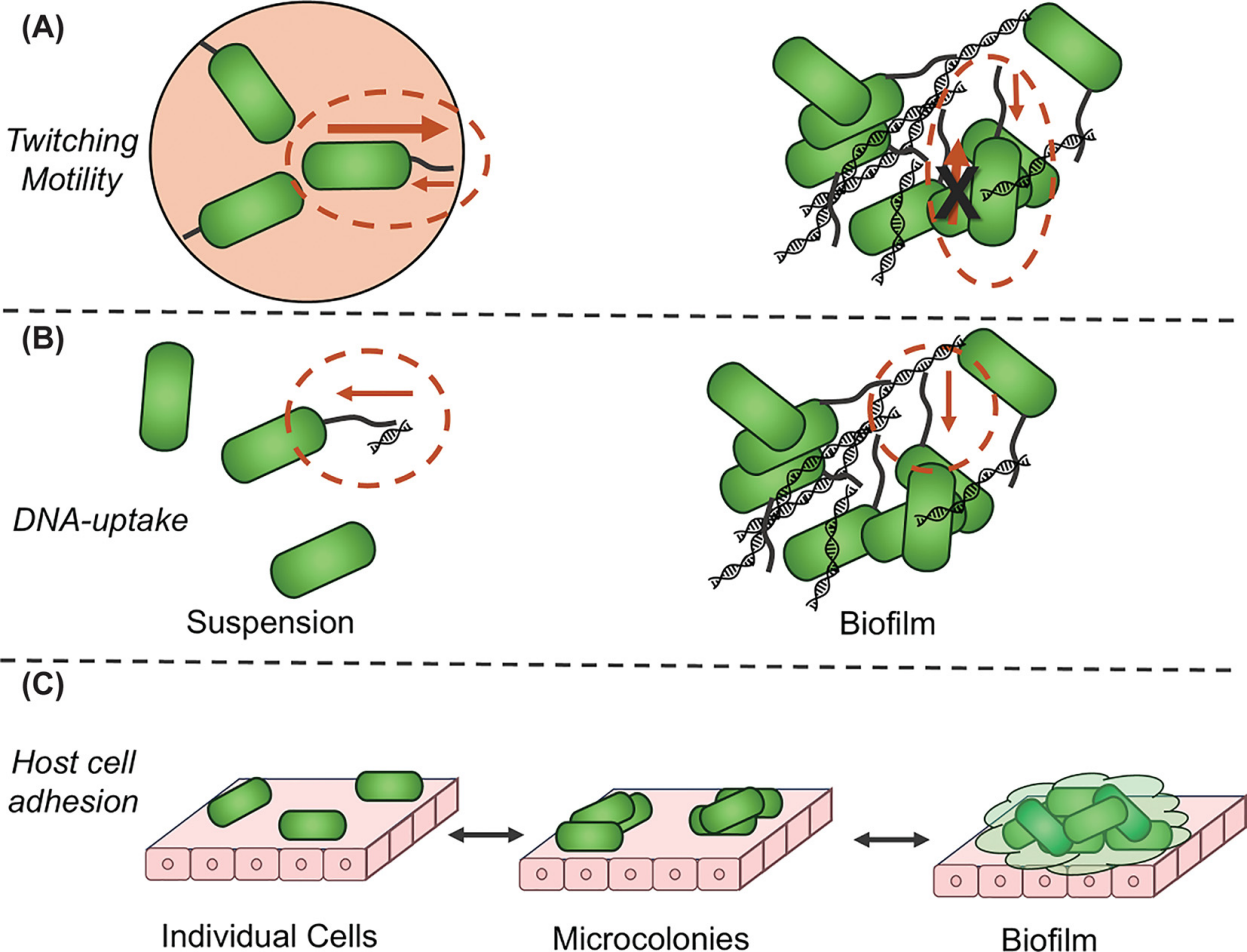
T4P functions in biofilms (A) Twitching motility, (B) DNA uptake, and (C) host-cell adhesion by single bacteria and bacterial aggregates. Cells are depicted in green and T4P and eDNA in black. (**A**) The cells highlighted in dashed orange are attached to an environmental surface and are either capable of motility if free or inhibited from movement by attachment (in biofilm). (**B**) The cells highlighted in dashed orange have T4P attached to eDNA and are potentially able to take it up in suspension or biofilm. (**C**) Individual cells transition to microcolonies through clonal expansion and then to biofilms, where an extracellular matrix (light green) is produced and cells begin to differentiate (shown with darker green cells).

Twitching motility is a specific type of surface motility, which is the result of T4P extending, adhering nonspecifically to a biotic or abiotic surface, and being retracted with sufficient force to pull a bacterial cell across the surface (Figure 3A) [28,52]. Unlike other forms of surface motility, twitching motility can occur at the interface of two surfaces so long as one of them is elastic. For this reason, assays specifically measuring twitching motility generally inoculate bacteria at the interface of nutrient agar and plastic [53]. The elasticity of ∼1%–2% agar is generally thought to be equivalent to biological tissues [54]. Because twitching motility occurs only on surfaces and cannot occur in suspension, it may represent one evolutionary factor increasing expression of T4P for surface-attached bacteria [43] and the inverse regulation of T4P and flagella [55,56]. Twitching motility may be a factor, along with clonal expansion, in the development of biofilms [57]. However, in the biofilm environment, bacterial cells are generally thought to be immobile, and several pieces of functional data suggest that T4P expression with little or no retraction may promote biofilm to a greater extent than T4P with greater retraction; multiple groups have reported that mutants of retraction ATPases are hyper-piliated and/or show enhanced biofilm [33,58,59]. Our group has also shown recently that naturally occurring defects in retraction contribute to differences in average pilus expression and *in vitro* biofilm formation [60].

### DNA uptake

Like twitching motility, DNA uptake by T4P is a three-step process that utilizes all of the T4P general mechanisms: extension, adhesion, and retraction (see Figure 3B). However, the specifics are reversed in two respects; in place of a non-specific surface attachment, DNA uptake requires attachment to DNA, and in some cases (e.g., *N. meningitidis*), there is demonstrable sequence specificity [23,24]. The orientation of movement during retraction is also reversed with the T4P pulling DNA cargo toward the cell rather than the bacterial cell being pulled toward the attachment point(s) of the pilus. DNA uptake can occur in suspension [60,61], but is frequently assayed on solid media, which promotes T4P expression [58]. The environmental conditions of biofilms also appear to promote DNA uptake in some cases; *P. aeruginosa*, which expresses T4P robustly under numerous conditions, does not take up eDNA under conditions where *Pseudomonas stutzeri* will [62] but does show natural competence in an *in vitro* model of biofilm [63]. The biofilm matrix may physically sequester DNA, increasing the concentration of DNA in biofilms relative to the areas outside of the biofilm and hence promoting the binding of T4P-incorporated DNA receptors. Transcriptional changes in biofilm cells may also promote the expression of natural competence proteins, not only T4P components but also the ComEC channel, ComEA, RecA, and others.

## Promotion of biofilm through T4P adhesion

In addition to the retraction-dependent functions described above, T4P can fundamentally alter bacterial physiology through T4P-mediated adhesion. Here, we describe potential mechanisms for the promotion of biofilms, *in vivo* and *in vitro*, through increased adhesion to biotic and abiotic surfaces, components of the extracellular matrix, and T4P themselves.

### Interactions with biotic surfaces

T4P mutants have shown defective adhesion to host cells for a variety of bacterial pathogens in both plants [64] and mammals [32,65–67], with pilus-associated proteins at the tip (*Pseudomonas* PilY or *Neisseria* PilC) being implicated [34]. Some predatory bacteria also use T4P as a means to adhere to their prey [68], indicating that at least some T4P also adhere to bacterial cells directly. Bacterial adhesion through T4P in nature can occur at any point along a gradient from individual bacteria through microcolonies to fully fledged biofilms (Figure 3C). However, the simultaneous ability of many T4P systems to mediate direct host cell adhesion and to promote adhesion indirectly through aggregation makes disentangling these two mechanisms difficult experimentally [69].

Experimental assays of host adhesion commonly combine bacteria in suspension with cell monolayers for relatively short periods (∼1 h) [32,65]. Because of the shorter timescale (when compared with assays of biofilm formation), these assays typically allow for only limited bacteria self-association. And for some species (*Pseudomonas*, *Acinetobacter*), the majority of adherent bacteria appear to be individual cells [32,65], while for others (*Neisseria*), the bacterial cells form microscopically visible microcolonies [70].

The T4P of the cellulose-degrading bacterium *Ruminococcus albus* was found to bind to cellulose directly, and a non-piliated mutant was defective for cellulose binding [71,72]. T4P expression has also been shown for *R. flavefaciens* [73], implying that adhesion through T4P could be a general mechanism for cellulolytic bacteria. Among Gram-positive non-pathogenic gastrointestinal (GI) bacterial isolates, T4P gene clusters were commonly found in genomes also possessing consortia of carbohydrate active enzymes [74]. Adhesion to polysaccharides represents another avenue for both host adhesion (particularly in plants) as well as a potential mechanism for interactions between T4P and the extracellular matrix in biofilms.

### Surface-sensing by T4P

T4P may also mediate transitory interactions with surfaces that allow for changes in cellular phenotype [75,76], including the expression of other more permeant adhesins. *Caulobacter crecentus* exhibits a biphasic life cycle between non-motile stalked cells and motile swarmer cells, with only the former being reproductive [77]. Upon cell division, swarmer cells will bud off the parent stalked cell and utilize a polar flagellum and co-located Tad pili for motility and surface adherence, respectively. In their biofilm forming cascade, the Tad pilus facilitates initial reversible adherence, anchoring cells perpendicular to their surface by their polar end [78]. In this state they can exhibit walking motility or proceed further to irreversible attachment and differentiation. This transition is marked by a spike in cellular cyclic di-guanosine mono phosphate (c-di-GMP) levels in the cell, which is primarily modulated through the diguanylate cyclase PleD, which is initially inactivated by PleC [79,80]. PleC embeds in the membrane proximal to the polar Tad pili and acts as a phosphatase. Eventually, it will either delocalize from the pole to be replaced with kinase DivJ or shift to exhibit kinase activity [77].

A number of mechanisms of mechano-sensing have been shown to regulate this process: the major pilin of the Tad pilus, PilA, was identified as the primary regulator of PleC in its conversion from phosphatase to kinase activity, where its transmembrane α-helix contains a cell-cycle initiating peptide sequence that interacts with PleC, suggesting that surface sensing is achieved through retraction of the Tad pili and reintroduction of polymerized PilA into the membrane pool [81]. Tad pili also engage in a parallel mechanism of surface sensing to stimulate differentiation upon obstruction of retraction, which stimulates delocalization of PleC and localization of DivJ, thereby allowing for surface sensing under conditions where surface adherence is achieved and complete retraction is blocked [82,83]. Surface sensing is also facilitated by the polar flagellum; the flagellar motor has been identified as an activator of an alternative diguanylate cyclase DgcB and may function as a sensor channel upon flagellar obstruction [84].

After initial adherence and stimulation of c-di-GMP synthesis, *C. crecentus* undergoes differentiation into stalked cells [77]. Several processes are stimulated, including genome replication and preparations for cell division and development of the stalked phenotype. This cascade also triggers the synthesis of a holdfast, a polysaccharide adhesin that irreversibly binds to the surface [83,85]. Holdfast adherence is key to development and growth of the biofilm, with a mediator of biofilm dispersal being the release of eDNA, which prevents holdfast adhesion of new swarmer cells [77]. In conditions favorable to biofilms, rapid adherence of Tad pili by the newly formed swarmer cell to the surface allows for microcolonies and larger cellular aggregates to form [84].

In *P. aeruginosa*, T4P also function as mechanosensors that enable the bacteria to detect surfaces through T4P retraction, allowing cells to interact with surfaces of various stiffness [86–88]. The mechanosensing mechanism is dependent upon the Pil-Chp chemosensory system, which includes chemotaxis array components that enable it to detect environmental signals from surface contact and mechanical input [89]. When T4P of *P. aeruginosa* attach to a surface and retract, tension develops in the pilus filaments, which may occur through multiple conformational equilibria [90]. PilY1, a non-pilin, pilus-associated protein in the tip complex of Pa T4P, contains a mechanosensitive von Willebrand factor A (VWFa) domain [91]. This tension travels through the pilus fiber to reach the chemoreceptor-like protein PilJ. This interaction leads to adenylate cyclase activation through CyaB, which results in increased intracellular cyclic adenosine mono phosphate (cAMP) levels. The cAMP-binding regulator Vfr controls the expression of virulence factors through this mechanism. The Chp system detects environmental signals, which trigger CyaB activation to produce cAMP. This process activates pilus genes through the Vfr pathway. T4P mechanosensing and chemosensing of extracellular polysaccharide [92] also initiate c-di-GMP production and c-di-GMP-dependent gene expression through multiple signaling pathways that control bacterial behavior on surfaces [93,94].

### T4P adherence to eDNA

As discussed above, numerous T4P systems incorporate DNA-binding subunits and have been known to bind DNA for decades as a prerequisite step in DNA uptake and natural competence. Interactions between T4P and longer fragments of eDNA also have the potential to stabilize multicellular assemblies through adherence to extracellular matrices of genomic DNA. Because multiple mechanisms exist by which T4P could plausibly promote biofilm formation (discussed in detail below), separating the effects of DNA-binding can be difficult experimentally. However, for T4P systems that incorporate DNA-binding subunits that are not essential for pilus biogenesis, mutants of those genes provide a useful comparison point. The discovery of DNA-binding subunits ComP (in *Neisseria*) [23] and FimT (in *Legionella*, *Pseudomonas*, *Acinetobacter*, and possibly many others) [26] was possible in part because those subunits are incorporated into initiation (tip) complexes but are not required for the initiation of biogenesis, potentially because other subunits can occupy that same position in the assembly.

**Figure 4:**
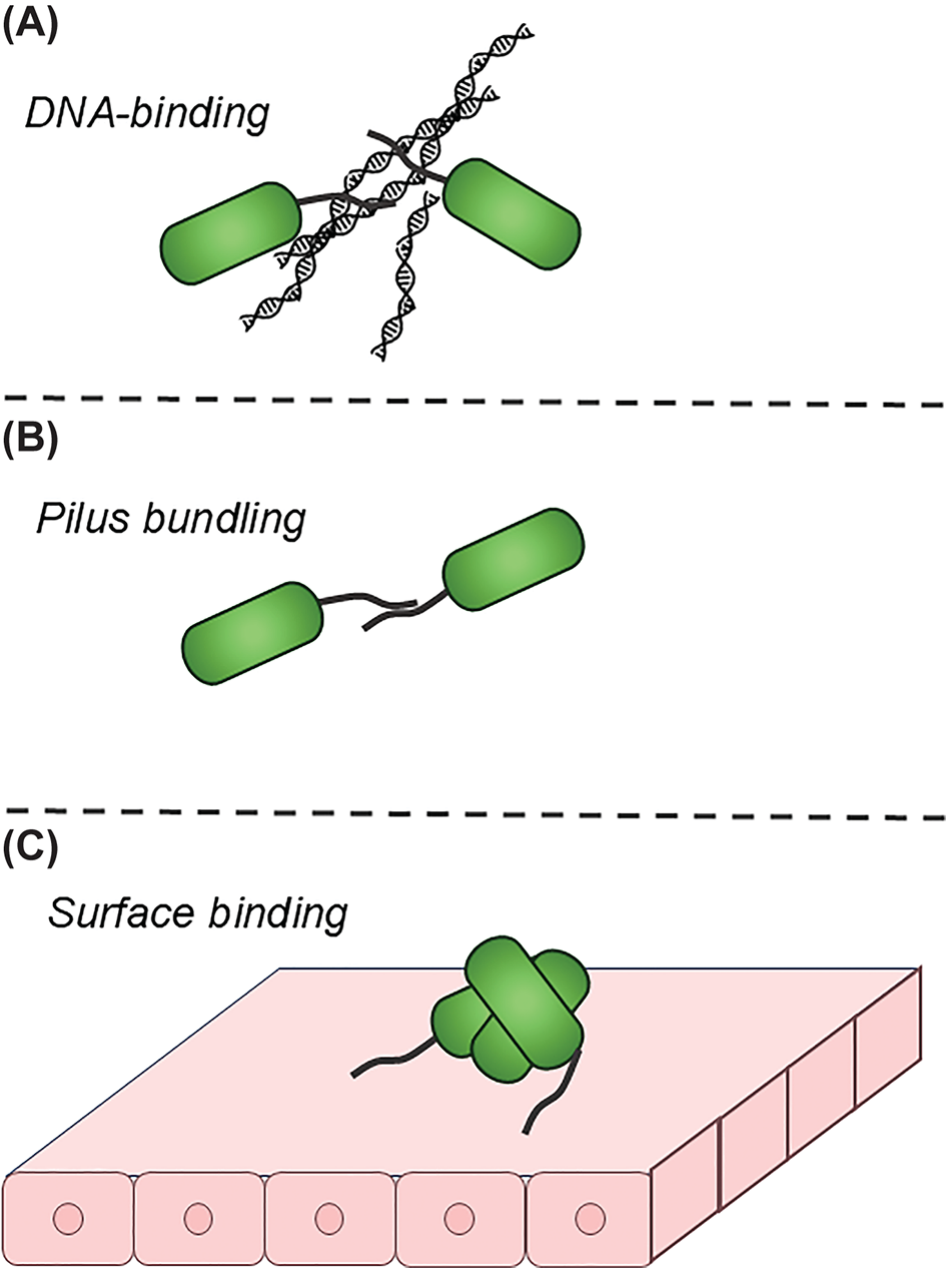
Mechanisms of T4P-mediated aggregation Potential T4P-mediated contributions to biofilm formation from (**A**) DNA-binding by T4P, (**B**) T4P bundling interactions in *trans*, and (**C**) adhesion by T4P to biotic surfaces.

From the *C. difficile* T4P system, we also have growing evidence that T4P DNA-binding subunits can specifically promote biofilm formation (see Figure 4A); DNA-binding subunits PilJ and PilW are differentially up-regulated in biofilms to a greater degree than the major pilus subunit PilA1 [95,96]. Mutations to *pilJ* and *pilW* also reduce *in vitro* biofilm formation, but neither to the degree of the *pilA1* mutant [25], supporting the notion that these subunits are not required for efficient pilus biogenesis, which is expected for subunits incorporated sporadically along the pilus fiber rather than as part of the initiation complex.

### T4P self-interactions

In addition to interactions with polysaccharides and eDNA, T4P can stabilize multicellular structures by interacting with another extracellular fiber, themselves. Numerous transmission electron microscopy experiments have shown bundles of T4P, either produced by the same cell (*cis*-bundles) or from neighboring cells (*trans*-bundles) [11,97]. These bundles of T4P can also be observed by SEM far more easily than single pili because of the narrow width of a single pilus, 6-9 nm [98], and *cis*-pilus bundles may be necessary in some cases for forceful retraction [99,100]. Bundling by T4P *in trans* is the proposed mechanism for T4P-mediated aggregation of EPEC through the bundle-forming pilus [101–103] and for *V. cholerae* through the toxin-coregulated pilus (see more below) [104] and was proposed by our group as a potential mechanism for biofilm formation in *Acinetobacter baumannii* [37], although more recent data favors other mechanisms, particularly naturally occurring differences in pilus retraction as described above under ‘T4P retraction in bacterial biofilms’ [60]. Figure 4B, shows pilus bundling *in trans* as a potential mechanism for cell-cell adhesion.

The mechanisms of pilus bundling are difficult to elucidate through structural biology because of the intractability of pilus bundles, but a role for specialized subunits was proposed in *Neisseria* through PilX, a pilin subunit incorporated into Neisseria T4P [98], which is not required for piliation but promotes aggregation into microcolonies on the host cell surface [66]. PilX contains an extended ‘hook’ at the C-terminus, which is necessary for its function, potentially through interactions between PilX subunits in *trans* pilus bundles [105].

## Aggregation in suspension

The term biofilm is typically used to describe multicellular, differentiated bacterial assemblies on surfaces [106], but the process of bacterial aggregation can occur both in suspension and on surfaces [107]. One can view these two processes as the development of both suspended and surface-attached biofilms or as aggregation in suspension and surface aggregation, but in either case, these are parallel processes with pronounced similarities. The molecular mechanisms underpinning bacterial aggregation appear to be held in common in some cases, including T4P, which can contribute to both. In continuous flow models of biofilm formation, T4P are required for the formation of ‘streamers,’ which can result in rapid clogging of microfluidic chambers [108]; T4P mutants also show defects in the general accumulation of biofilm on the chamber surfaces of these models [31].

**Figure 5:**
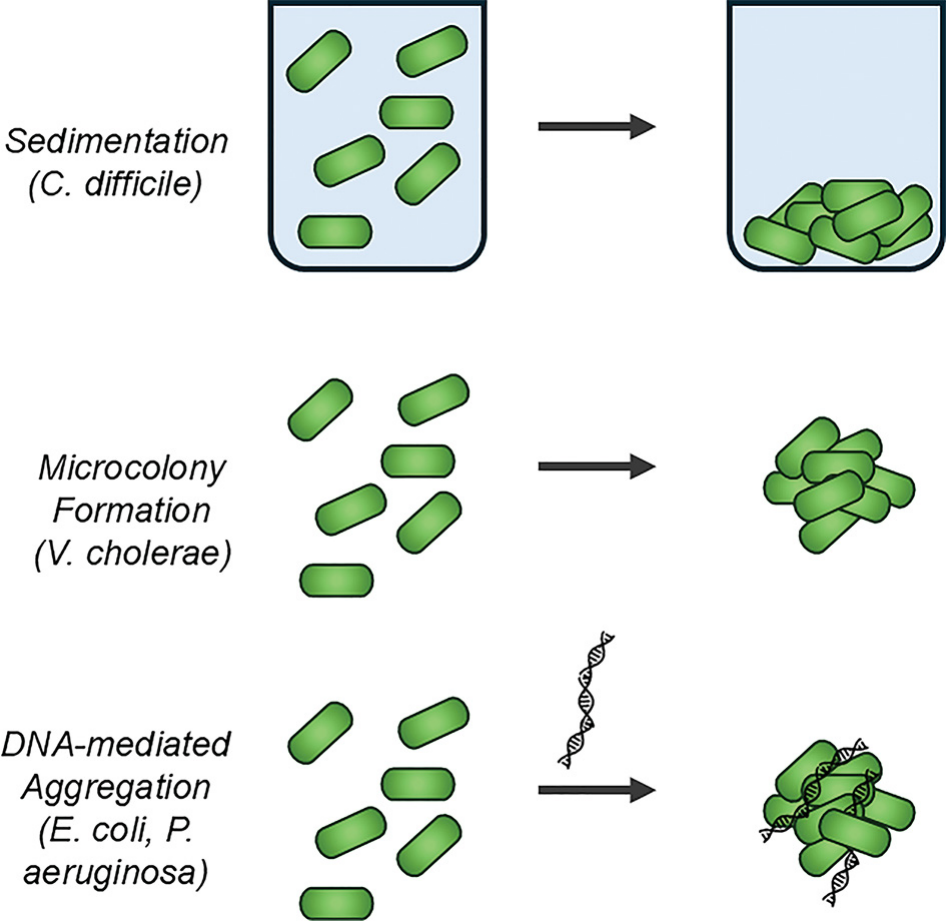
Models of biofilm in bacterial suspension Schematic representations of (i) sedimentation/aggregation in bacterial cultures, (ii) microcolony formation in suspension, and (iii) DNA-mediated aggregation.

The TCP of *V. cholerae* is one of the best-characterized examples of T4P promoting bacterial aggregation in suspension; the TCP is required for the formation of microcolonies (see Figure 5), which are able to pass through the host stomach (where individual *V. cholerae* cells cannot survive) into the intestines where *V. cholerae* can expand and secrete toxins, including Cholera Toxin (CT) [109]. This process is an essential step in pathogenesis, and without the TCP, *V. cholerae* is noninfectious in mouse models of cholera [104]. In experimental models of *V. cholerae* biofilm on chitinous surfaces, the TCP- mutants were also defective [110], which can be explained in terms of a T4P-mediated microcolony formation as a common molecular mechanism for both surface-attached and suspended aggregation.

In *C. difficile*, T4P mutants also show clear defects in both assays of auto-aggregation [54,56] (see Figure 5) and surface biofilm formation [25,95]. As discussed above, some of this activity could be explained through specific adhesion to eDNA [25]. On the face of it, this mechanism is more appropriate to surface biofilms than in suspension, where eDNA molecules could presumably diffuse away. However, multiple groups have found significant differences in aggregation in suspension with the addition of exogenous DNA [111–113] (see Figure 5), and this capacity appears to be both generalizable and controllable [114]. *Clostridioides difficile* T4P genes are more highly expressed in surface biofilms and colony biofilms than in suspension [95], but if the suspension includes a mixture of individual cells and microcolonies, the microcolonies may also show increased expression with the average reduced by the individual cells.

## Targeting T4P as an anti-biofilm strategy

Although T4P are required for biofilm formation and (in many cases) for the virulence of bacterial pathogens [115], no T4P gene has ever been found to be required for survival. This combination makes targeting T4P biogenesis or function a potential therapeutic avenue to combat bacterial virulence without creating evolutionary pressure for the rapid development of antimicrobial resistance [116]. As an evolutionary hypothesis, antivirulence drugs should provoke resistance more slowly than their toxic counterparts [117–120]. Unlike highly bacteriotoxic compounds, which would leave behind only resistant, virulent bacterial cells, interventions that reduce virulence would create populations where resistant, virulent cells would compete with non-resistant, avirulent cells for resources [121]. To date, T4P-targeting drugs, vaccines, and therapeutic antibodies have been employed against bacterial pathogens, and each of these approaches has potential advantages, which we discuss here.

### Next-generation antimicrobials targeting pilus biogenesis and dynamics

As discussed above, T4P biogenesis requires extension through the action of a cytoplasmic AAA+ ATPase, generally referred to as PilB (*P. aeruginosa, A. baumannii, C. difficile*) or PilF (*N. meningitidis, T. thermophilus*). This process requires the formation of a hexameric ring of enzymatic subunits and coordinated conformational changes that rotate the ring and the pilus fiber relative to the membrane [122]. A homologous enzyme (PilT, or a complex of PilT and PilU) catalyzes the reverse reaction. Inhibition of pilus biogenesis and retraction can be measured easily through functional assays of twitching motility [31] or microcolony formation [70], phage uptake by T4P-targeting viruses [123], or fluorescence microscopy using cells expressing tagged pilin proteins [124].

Compounds inhibiting T4P biogenesis were first discovered using a screen of FDA-approved drugs to inhibit microcolony formation in *N. meningitidis* [70]. These compounds were phenothiazines, a class of compounds with a history of use as stains (methylene blue) and as antimicrobials [125–128] but approved for treatment of psychiatric disorders [129,130]. Two compounds, thioridazine and trifluoperazine, were found to be effective in reducing the formation of *Neisseria* microcolonies *in vitro*. Thioridazine also improved survival in a mouse infection model, on its own or in combination with Cefotaxime [70]. The mechanism of action is T4P-dependent, and these compounds inhibit piliation measured by fluorescence microscopy using an anti-T4P fluorescent antibody. These compounds were ineffective against a *pilT* mutant, suggesting that these compounds affect later pilus extension/retraction dynamics rather than inhibiting pilus biogenesis. Our group found that these same compounds were effective in inhibiting T4P functions, including twitching motility and biofilm formation in *A. baumannii* and *P. aeruginosa*, implying a broad applicability against gammaproteobacteria [31]. However, the MICs for these effects are high (∼low μM), and for some related species (including *Moraxella bovoculi*), we observed cytotoxicity in a similar concentration range.

An alternative and complementary approach is to target T4P biogenesis directly by inhibiting the extension of T4P. A high-throughput screen of using an *in vitro* assay of PilB ATPase activity found two compounds, benserazide and levodopa. These are structurally related drugs approved for Parkinson’s disease and both show activity against PilB directly and in inhibiting *Myxococcus xanthus* twitching motility and biofilm formation [131]. Additional studies expanded upon these methods, including computational screening for additional lead compounds [132] and the use of T4P-specific phages to identify T4P-targeting compounds in *P. aeruginosa* [123]. The latter study resulted in the discovery of anti-T4P activity for tuspetinib, which inhibits twitching motility in *P. aeruginosa* and *A. baumannii* without appreciable cytotoxicity [123]. Because of the relationships between T4P in biofilms conserved across many bacterial taxa, these high-throughput approaches are likely broadly applicable [133]. However, for the specific inhibition of biofilm, both twitching motility and phage vulnerability are complicated because they depend on pilus retraction, which is not necessarily required for biofilm formation as discussed above.

### Therapeutic antibodies targeting T4P

Several of the experimental approaches described above use monoclonal antibodies or antisera against pilin subunits, and in broad strokes, T4P subunits are immunogenic [134–136]. Antibody binding was found to disrupt pilus retraction in assays of twitching motility and phage infection, leading to the understanding that those processes were retraction-dependent [28,137]. Several studies have also used anti-T4P antibodies to deliberately disrupt T4P functions, including biofilm formation. Antibodies against the major T4P subunit (PilA) in *Haemophilus influenzae* are able to disperse single-species biofilms [138] and mixed biofilms of *H. influenzae* and *Moraxella catarrhalis* [139] and several other bacterial species [140]. There is also one recent report that therapeutic antibodies can provide protection against *P. aeruginosa* in a lung infection model [141].

### Pilins as vaccine components

T4P are composed of 1000s of subunits, completely exposed and extended micrometers out from bacteria cells, and, as discussed above, immunogenic. As early as the general structure of T4P was understood, they were thought of as obvious vaccine targets. Because the major subunit is orders of magnitude more highly expressed than the others, it was the initial target of several vaccines, in spite of the high level of variability in many species [38]. To combat this variability, the earliest vaccines used multiple gene products; a vaccine against *Dichelobacter nodosus* in sheep used a combination of nine naturally occurring variants of the major pilin (FimA) [142]. These multivalent vaccines are still in use in some countries but are generally considered to be less effective than monovalent or bivalent vaccines [143], which require serotyping for effective selection [144]. As an alternative method, a recombinant pilin protein using a consensus sequence of six *P. aeruginosa* PilA proteins was used as a vaccine, which showed some effectiveness in a murine model of acute infection [145,146]. Vaccines targeting specific strains showed effectiveness in several cases [147–149], but other studies have shown that vaccination with pilin proteins is insufficiently immunogenic [150] or fails to provide protection despite high titers of anti-T4P antibodies [151,152].

**Figure 6:**
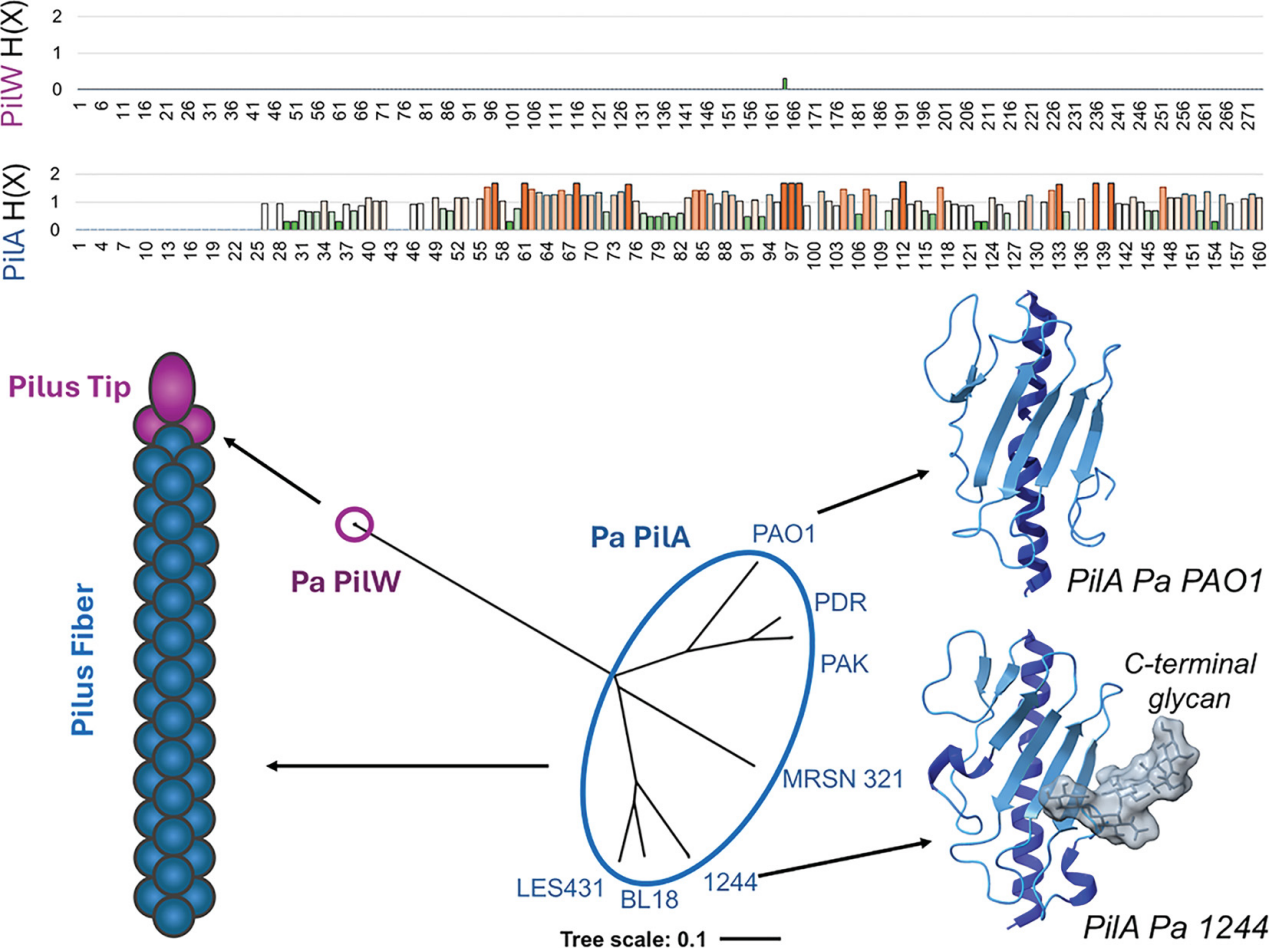
Relative conservation of pilin subunits and suitability as antigens (Above) Shannon entropy H(*x*) plots for alignments of PilA (major pilin) and PilW (part of the tip complex) for 11 strains of *P. aeruginosa*. Alignment positions in the H(*x*) plot are colored from dark green (low entropy) to orange (high entropy) (below). Phylogram of *P. aeruginosa* PilA and PilW sequences aligned with Clustal Omega [157] and represented using the Interactive Tree Of Life (iTOL) [158]. Two structures of PilA proteins (PDB IDs, 6BBK and 8TUW, [159]) showing the divergence, which inhibits the development of broad protective immunity from PilA vaccines.

These negative results could result in part from high levels of variability in the major subunit. Figure 6 shows Shannon entropy plots and phylodendrograms for two gene products from *P. aeruginosa*, PilA (the major subunit) and PilW (a minor subunit incorporated into the tip complex). Both the H(X) plots and the dendrogram branch length show that Pa PilA is much more variable than Pa PilW. Many bacterial species also incorporate specific glycosylation sites into the major pilin, either at the C-terminus [153] or other regions of the exposed C-terminal domain [154], as shown in Figure 6 for Pa PilA (strain PAO1), which is not terminally glycosylated, and Pa PilA (strain 1244), which is. Because of the relatively high conservation of minor pilin subunits, we propose that they may show higher potential than the primary subunits as vaccine candidates. Broad protection from a pilin subunit vaccine may be more achievable using members of the tip complex as antigens, or intercalated pilins, as they may provide a greater number of conserved surfaces than the initiation complex found only at the tip [11,35,45].

## Conclusions

T4P and their subunits have been described as prokaryotic Swiss army knives [1] and versatile molecular modules [44] because of their incredible adaptability to a wide variety of physiological functions based on three processes: extension, adhesion, and retraction. Each of these processes is impacted by the microenvironments within bacterial biofilms; T4P biogenesis is up-regulated in biofilms, adhesion is impacted by vastly different concentrations of T4P ligands, including eDNA and possibly the T4P themselves, and retraction-mediated motility may be limited by special constraints. To understand this bi-directional relationship between T4P function and biofilm formation, determining the specific adhesive properties of T4P subunits may be a path forward. In particular, the potential of T4P fibers to adhere bacteria to surfaces directly and to create surface attachment indirectly by cross-linking bacterial cells makes differentiating host adhesion and biofilm formation particularly difficult. As the properties of specific T4P subunits are elucidated, more specific genetic alterations will allow for better controlled experiments. The inhibition or dispersal of bacterial biofilms through inhibition of T4P biogenesis is rapidly developing as a complementary approach for the treatment of bacterial infections. We anticipate that the study of T4P tip complexes and intercalated pilins will also provide an avenue for the development of T4P as effective vaccine candidates.

## Perspectives

- Bacterial biofilms are of wide and increasing interest because of their roles in infectious disease and antimicrobial resistance. The mechanisms of type IV pilus-mediated biofilm formation are of particular interest because of the diversity of T4P fibers and their use by clinically important pathogens, including ESKAPE pathogens *P. aeruginosa* and *A. baumannii*.
- Bacterial aggregation is a core component of biofilm formation, and the importance of bacterial adhesion to eDNA (including by T4P) during bacterial aggregation has been reinforced by discoveries in multiple species.
- Because of their use by a variety of bacterial taxa, T4P continue to make attractive targets for interventions, but the focus in recent years has shifted from T4P major subunits as vaccine candidates to drugs targeting pilus biogenesis. We anticipate that these trends will continue but that T4P components in the tip complexes and conserved intercalated pilin subunits could become attractive antigenic targets for vaccines or therapeutic antibodies.

## Open Access

Open access for this article was enabled by the participation of the University of Nebraska, Lincoln in an all-inclusive Read & Publish agreement with Portland Press and the Biochemical Society under an agreement with the Big Ten Alliance.

## Abbreviations

c-di-GMP: cyclic di-guanosine mono phosphate
cAMP: cyclic adenosine mono phosphate
eDNA: extracellular DNA
EPEC: enteropathogenic *E. coli*
T2SS: type II secretion system
T4P: type IV pili
TCP: toxin-corregulated pilus

## References

[1] Berry J.L. and Pelicic V. (2015) Exceptionally widespread nanomachines composed of type IV pilins: the prokaryotic Swiss Army knives. FEMS Microbiol. Rev. 39, 134–154 10.1093/femsre/fuu001 25793961 PMC4471445

[2] Denise R., Abby S.S. and Rocha E.P.C. (2019) Diversification of the type IV filament superfamily into machines for adhesion, protein secretion, DNA uptake, and motility. PLoS Biol. 17, e3000390 10.1371/journal.pbio.3000390 31323028 PMC6668835

[3] Piepenbrink K.H. (2019) DNA uptake by Type IV filaments. Front. Mol. Biosci. 6, 1 10.3389/fmolb.2019.00001 30805346 PMC6370729

[4] Sheppard D., Berry J.L., Denise R., Rocha E.P.C., Matthews S. and Pelicic V. (2020) The major subunit of widespread competence pili exhibits a novel and conserved type IV pilin fold. J. Biol. Chem. 295, 6594–6604 10.1074/jbc.RA120.013316 32273343 PMC7212644

[5] Whitfield G.B. and Brun Y.V. (2024) The type IVc pilus: just a Tad different. Curr. Opin. Microbiol. 79, 102468 10.1016/j.mib.2024.102468 38579360

[6] Dazzoni R., Li Y., Lopez-Castilla A., Brier S., Mechaly A., Cordier F. et al. (2023) Structure and dynamic association of an assembly platform subcomplex of the bacterial type II secretion system. Structure 31, 152e157–165e157 10.1016/j.str.2022.12.003 36586404

[7] Li Y., Santos-Moreno J. and Francetic O. (2023) The periplasmic coiled coil formed by the assembly platform proteins PulL and PulM is critical for function of the Klebsiella type II secretion system. Res. Microbiol. 174, 104075 10.1016/j.resmic.2023.104075 37141929

[8] Shaliutina-Loginova A., Francetic O. and Dolezal P. (2023) Bacterial type II secretion system and its mitochondrial counterpart. mBio 14, e0314522 10.1128/mbio.03145-22 36971557 PMC10128026

[9] Jarrell K.F. and Albers S.V. (2012) The archaellum: an old motility structure with a new name. Trends Microbiol. 20, 307–312 10.1016/j.tim.2012.04.007 22613456

[10] Shahapure R., Driessen R.P., Haurat M.F., Albers S.V. and Dame R.T. (2014) The archaellum: a rotating type IV pilus. Mol. Microbiol. 91, 716–723 10.1111/mmi.12486 24330313

[11] Piepenbrink K.H., Maldarelli G.A., Martinez de la Pena C.F., Dingle T.C., Mulvey G.L., Lee A. et al. (2015) Structural and evolutionary analyses show unique stabilization strategies in the type IV pili of *Clostridium difficile*. Structure 23, 385–396 10.1016/j.str.2014.11.018 25599642 PMC4318773

[12] Neuhaus A., Selvaraj M., Salzer R., Langer J.D., Kruse K., Kirchner L. et al. (2020) Cryo-electron microscopy reveals two distinct type IV pili assembled by the same bacterium. Nat. Commun. 11, 2231 10.1038/s41467-020-15650-w 32376942 PMC7203116

[13] Anger R., Pieulle L., Shahin M., Valette O., Le Guenno H., Kosta A. et al. (2023) Structure of a heteropolymeric type 4 pilus from a monoderm bacterium. Nat. Commun. 14, 7143 10.1038/s41467-023-42872-5 37932265 PMC10628169

[14] Bovre K. and Froholm L.O. (1971) Competence of genetic transformation correlated with the occurrence of fimbriae in three bacterial species. Nat. New Biol. 234, 151–152 10.1038/newbio234151a0 5289316

[15] Seifert H.S., Ajioka R.S., Paruchuri D., Heffron F. and So M. (1990) Shuttle mutagenesis of* Neisseria gonorrhoeae*: pilin null mutations lower DNA transformation competence. J. Bacteriol. 172, 40–46 10.1128/jb.172.1.40-46.1990 2152910 PMC208398

[16] Facius D., Fussenegger M. and Meyer T.F. (1996) Sequential action of factors involved in natural competence for transformation of *Neisseria gonorrhoeae*. FEMS Microbiol. Lett. 137, 159–164 10.1111/j.1574-6968.1996.tb08099.x 8998979

[17] Porstendörfer D., Drotschmann U. and Averhoff B. (1997) A novel competence gene, comP, is essential for natural transformation of *Acinetobacter* sp. strain BD413. Appl. Environ. Microbiol. 63, 4150–4157 10.1128/aem.63.11.4150-4157.1997 9361398 PMC168731

[18] Griffith F. (1928) The significance of pneumococcal types. J. Hyg. (Lond.) 27, 113–159 10.1017/S0022172400031879 20474956 PMC2167760

[19] Fussenegger M., Rudel T., Barten R., Ryll R. and Meyer T.F. (1997) Transformation competence and type-4 pilus biogenesis in *Neisseria gonorrhoeae*—a review. Gene 192, 125–134 10.1016/S0378-1119(97)00038-3 9224882

[20] Meibom K.L., Blokesch M., Dolganov N.A., Wu C.Y. and Schoolnik G.K. (2005) Chitin induces natural competence in *Vibrio cholerae*. Science 310, 1824–1827 10.1126/science.1120096 16357262

[21] Hulter N., Sorum V., Borch-Pedersen K., Liljegren M.M., Utnes A.L., Primicerio R. et al. (2017) Costs and benefits of natural transformation in *Acinetobacter baylyi*. BMC Microbiol. 17, 34 10.1186/s12866-017-0953-2 28202049 PMC5312590

[22] VanWagoner T.M., Whitby P.W., Morton D.J., Seale T.W. and Stull T.L. (2004) Characterization of three new competence-regulated operons in *Haemophilus influenzae*. J. Bacteriol. 186, 6409–6421 10.1128/JB.186.19.6409-6421.2004 15375121 PMC516621

[23] Cehovin A., Simpson P.J., McDowell M.A., Brown D.R., Noschese R., Pallett M. et al. (2013) Specific DNA recognition mediated by a type IV pilin. Proc. Natl. Acad. Sci. U.S.A. 110, 3065–3070 10.1073/pnas.1218832110 23386723 PMC3581936

[24] Berry J.L., Xu Y., Ward P.N., Lea S.M., Matthews S.J. and Pelicic V. (2016) A comparative structure/function analysis of two type IV pilin DNA receptors defines a novel mode of DNA binding. Structure 24, 926–934 10.1016/j.str.2016.04.001 27161979 PMC4906244

[25] Ronish L.A., Sidner B., Yu Y. and Piepenbrink K.H. (2022) Recognition of extracellular DNA by type IV pili promotes biofilm formation by *Clostridioides difficile*. J. Biol. Chem. 298, 102449 10.1016/j.jbc.2022.102449 36064001 PMC9556784

[26] Braus S.A.G., Short F.L., Holz S., Stedman M.J.M., Gossert A.D. and Hospenthal M.K. (2022) The molecular basis of FimT-mediated DNA uptake during bacterial natural transformation. Nat. Commun. 13, 1065 10.1038/s41467-022-28690-1 35246533 PMC8897410

[27] Salleh M.Z., Karuppiah V., Snee M., Thistlethwaite A., Levy C.W., Knight D. et al. (2019) Structure and properties of a natural competence-associated pilin suggest a unique pilus tip-associated DNA receptor. mBio. 10, e00614–19 10.1128/mBio.00614-19 31186316 PMC6561018

[28] Bradley D.E. (1980) A function of *Pseudomonas aeruginosa* PAO polar pili: twitching motility. Can. J. Microbiol. 26, 146–154 10.1139/m80-022 6105908

[29] Henrichsen J. and Blom J. (1975) Correlation between twitching motility and possession of polar fimbriae in *Acinetobacter calcoaceticus*. Acta Pathol. Microbiol. Scand. B. 83, 103–115 1155109 10.1111/j.1699-0463.1975.tb00078.x

[30] O'Hara M.T., Shimozono T.M., Dye K.J., Harris D. and Yang Z. (2024) Surface hydrophilicity promotes bacterial twitching motility. mSphere 9, e0039024 10.1128/msphere.00390-24 39194233 PMC11423576

[31] Vo N., Sidner B.S., Yu Y. and Piepenbrink K.H. (2023) Type IV pilus-mediated inhibition of *Acinetobacter baumannii* biofilm formation by phenothiazine compounds. Microbiol. Spectr. 11, e0102323 10.1128/spectrum.01023-23 37341603 PMC10433872

[32] Piepenbrink K.H., Lillehoj E., Harding C.M., Labonte J.W., Zuo X., Rapp C.A. et al. (2016) Structural diversity in the type IV pili of multidrug-resistant Acinetobacter. J. Biol. Chem. 291, 22924–22935 10.1074/jbc.M116.751099 27634041 PMC5087714

[33] Chiang P. and Burrows L.L. (2003) Biofilm formation by hyperpiliated mutants of *Pseudomonas aeruginosa*. J. Bacteriol. 185, 2374–2378 10.1128/JB.185.7.2374-2378.2003 12644510 PMC151504

[34] Nguyen Y., Sugiman-Marangos S., Harvey H., Bell S.D., Charlton C.L., Junop M.S. et al. (2015) *Pseudomonas aeruginosa* minor pilins prime type IVa pilus assembly and promote surface display of the PilY1 adhesin. J. Biol. Chem. 290, 601–611 10.1074/jbc.M114.616904 25389296 PMC4281761

[35] Barnier J.P., Meyer J., Kolappan S., Bouzinba-Segard H., Gesbert G., Jamet A. et al. (2021) The minor pilin PilV provides a conserved adhesion site throughout the antigenically variable meningococcal type IV pilus. Proc. Natl. Acad. Sci. U.S.A. 118, 10.1073/pnas.2109364118 34725157 PMC8609321

[36] Giltner C.L., Habash M. and Burrows L.L. (2010) *Pseudomonas aeruginosa* minor pilins are incorporated into type IV pili. J. Mol. Biol. 398, 444–461 10.1016/j.jmb.2010.03.028 20338182

[37] Ronish L.A., Lillehoj E., Fields J.K., Sundberg E.J. and Piepenbrink K.H. (2019) The structure of PilA from *Acinetobacter baumannii* AB5075 suggests a mechanism for functional specialization in Acinetobacter type IV pili. J. Biol. Chem. 294, 218–230 10.1074/jbc.RA118.005814 30413536 PMC6322890

[38] Cehovin A., Winterbotham M., Lucidarme J., Borrow R., Tang C.M., Exley R.M. et al. (2010) Sequence conservation of pilus subunits in *Neisseria meningitidis*. Vaccine 28, 4817–4826 10.1016/j.vaccine.2010.04.065 20457291

[39] Korotkov K.V. and Hol W.G. (2008) Structure of the GspK-GspI-GspJ complex from the enterotoxigenic *Escherichia coli* type 2 secretion system. Nat. Struct. Mol. Biol. 15, 462–468 10.1038/nsmb.1426 18438417

[40] Ng D., Harn T., Altindal T., Kolappan S., Marles J.M., Lala R. et al. (2016) The *Vibrio cholerae* minor pilin TcpB initiates assembly and retraction of the toxin-coregulated pilus. PLoS Pathog. 12, e1006109 10.1371/journal.ppat.1006109 27992883 PMC5207764

[41] Raynaud C., Sheppard D., Berry J.L., Gurung I. and Pelicic V. (2021) PilB from *Streptococcus sanguinis* is a bimodular type IV pilin with a direct role in adhesion. Proc. Natl. Acad. Sci. U.S.A. 118, e2102092118 10.1073/pnas.2102092118 34031252 PMC8179133

[42] Craig L. and Li J. (2008) Type IV pili: paradoxes in form and function. Curr. Opin. Struct. Biol. 18, 267–277 10.1016/j.sbi.2007.12.009 18249533 PMC2442734

[43] Pelicic V. (2008) Type IV pili: e pluribus unum? Mol. Microbiol. 68, 827–837 10.1111/j.1365-2958.2008.06197.x 18399938

[44] Giltner C.L., Nguyen Y. and Burrows L.L. (2012) Type IV pilin proteins: versatile molecular modules. Microbiol. Mol. Biol. Rev. 76, 740–772 10.1128/MMBR.00035-12 23204365 PMC3510520

[45] Piepenbrink K.H., Maldarelli G.A., de la Pena C.F., Mulvey G.L., Snyder G.A., De Masi L. et al. (2014) Structure of *Clostridium difficile* PilJ exhibits unprecedented divergence from known type IV pilins. J. Biol. Chem. 289, 4334–4345 10.1074/jbc.M113.534404 24362261 PMC3924296

[46] Obergfell K.P. and Seifert H.S. (2016) The pilin N-terminal domain maintains *Neisseria gonorrhoeae* Transformation competence during pilus phase variation. PLos Genet. 12, e1006069 10.1371/journal.pgen.1006069 27213957 PMC4877100

[47] Gu Y., Srikanth V., Salazar-Morales A.I., Jain R., O'Brien J.P., Yi S.M. et al. (2021) Structure of* Geobacter* pili reveals secretory rather than nanowire behaviour. Nature 597, 430–434 10.1038/s41586-021-03857-w 34471289 PMC9127704

[48] Reardon P.N. and Mueller K.T. (2013) Structure of the type IVa major pilin from the electrically conductive bacterial nanowires of *Geobacter sulfurreducens*. J. Biol. Chem. 288, 29260–29266 10.1074/jbc.M113.498527 23965997 PMC3795228

[49] Sonani R.R., Sanchez J.C., Baumgardt J.K., Kundra S., Wright E.R., Craig L. et al. (2023) Tad and toxin-coregulated pilus structures reveal unexpected diversity in bacterial type IV pili. Proc. Natl. Acad. Sci. U.S.A. 120, e2316668120 10.1073/pnas.2316668120 38011558 PMC10710030

[50] Rudel T., Scheurerpflug I. and Meyer T.F. (1995) Neisseria PilC protein identified as type-4 pilus tip-located adhesin. Nature 373, 357–359 10.1038/373357a0 7830772

[51] Kolappan S., Ng D., Yang G., Harn T. and Craig L. (2015) Crystal structure of the minor pilin CofB, the initiator of CFA/III pilus assembly in enterotoxigenic *Escherichia coli*. J. Biol. Chem. 290, 25805–25818 10.1074/jbc.M115.676106 26324721 PMC4646235

[52] Henrichsen J. (1983) Twitching motility. Ann. Rev. Microbiol. 37, 81–93 10.1146/annurev.mi.37.100183.000501 6139059

[53] Biswas I., Machen A. and Mettlach J. (2019) *In vitro* motility assays for *Acinetobacter* species. Methods Mol. Biol. 1946, 177–187 10.1007/978-1-4939-9118-1_17 30798555

[54] Purcell E.B., McKee R.W., Bordeleau E., Burrus V. and Tamayo R. (2016) Regulation of type IV pili contributes to surface behaviors of historical and epidemic strains of *Clostridium difficile*. J. Bacteriol. 198, 565–577 10.1128/JB.00816-15 26598364 PMC4719444

[55] Purcell E.B., McKee R.W., McBride S.M., Waters C.M. and Tamayo R. (2012) Cyclic diguanylate inversely regulates motility and aggregation in *Clostridium difficile*. J. Bacteriol. 194, 3307–3316 10.1128/JB.00100-12 22522894 PMC3434733

[56] Bordeleau E., Purcell E.B., Lafontaine D.A., Fortier L.C., Tamayo R. and Burrus V. (2015) Cyclic di-GMP riboswitch-regulated type IV pili contribute to aggregation of *Clostridium difficile*. J. Bacteriol. 197, 819–832 10.1128/JB.02340-14 25512308 PMC4325102

[57] O'Toole G.A. and Kolter R. (1998) Flagellar and twitching motility are necessary for *Pseudomonas aeruginosa* biofilm development. Mol. Microbiol. 30, 295–304 10.1046/j.1365-2958.1998.01062.x 9791175

[58] Harding C.M., Tracy E.N., Carruthers M.D., Rather P.N., Actis L.A. and Munson R.S. (2013) Acinetobacter baumannii strain M2 produces type IV pili which play a role in natural transformation and twitching motility but not surface-associated motility. mBio 4, 00360–13 10.1128/mBio.00360-13 23919995 PMC3735195

[59] Carbonnelle E., Helaine S., Nassif X. and Pelicic V. (2006) A systematic genetic analysis in *Neisseria meningitidis* defines the Pil proteins required for assembly, functionality, stabilization and export of type IV pili. Mol. Microbiol. 61, 1510–1522 10.1111/j.1365-2958.2006.05341.x 16968224

[60] Yu Y., Mahdi R., Al-Hilfy Leon A., Vo N., Lofgren R., Mutabazi J.L. et al. (2025) Variation in type IV pilus stability modulates DNA uptake and biofilm formation. J. Biol. Chem. 301, 110787 10.1016/j.jbc.2025.110787 41062070 PMC12603735

[61] Godeux A.S., Lupo A., Haenni M., Guette-Marquet S., Wilharm G., Laaberki M.H. et al. (2018) Fluorescence-based detection of natural transformation in drug-resistant *Acinetobacter baumannii*. J. Bacteriol. 200, e00181–18 10.1128/JB.00181-18 30012729 PMC6148472

[62] Meier P., Berndt C., Weger N. and Wackernagel W. (2002) Natural transformation of *Pseudomonas stutzeri* by single-stranded DNA requires type IV pili, competence state and comA. FEMS Microbiol. Lett. 207, 75–80 10.1111/j.1574-6968.2002.tb11031.x 11886754

[63] Nolan L.M., Turnbull L., Katrib M., Osvath S.R., Losa D., Lazenby J.J. et al. (2020) *Pseudomonas aeruginosa* is capable of natural transformation in biofilms. Microbiology (Reading) 166, 995–1003 10.1099/mic.0.000956 32749953 PMC7660920

[64] Taguchi F. and Ichinose Y. (2011) Role of type IV pili in virulence of *Pseudomonas syringae* pv. tabaci 6605: correlation of motility, multidrug resistance, and HR-inducing activity on a nonhost plant. Mol. Plant Microbe Interact. 24, 1001–1011 10.1094/MPMI-02-11-0026 21615203

[65] Farinha M.A., Conway B.D., Glasier L.M., Ellert N.W., Irvin R.T., Sherburne R. et al. (1994) Alteration of the pilin adhesin of *Pseudomonas aeruginosa* PAO results in normal pilus biogenesis but a loss of adherence to human pneumocyte cells and decreased virulence in mice. Infect. Immun. 62, 4118–4123 10.1128/iai.62.10.4118-4123.1994 7927665 PMC303085

[66] Hélaine S., Carbonnelle E., Prouvensier L., Beretti J.L., Nassif X. and Pelicic V. (2005) PilX, a pilus-associated protein essential for bacterial aggregation, is a key to pilus-facilitated attachment of *Neisseria meningitidis* to human cells. Mol. Microbiol. 55, 65–77 10.1111/j.1365-2958.2004.04372.x 15612917

[67] McKee R.W., Aleksanyan N., Garrett E.M. and Tamayo R. (2018) Type IV pili promote *Clostridium difficile* adherence and persistence in a mouse model of infection. Infect. Immun. 86, e00943–17 10.1128/IAI.00943-17 29483294 PMC5913833

[68] Chanyi R.M. and Koval S.F. (2014) Role of type IV pili in predation by *Bdellovibrio bacteriovorus*. PloS One 9, e113404 10.1371/journal.pone.0113404 25409535 PMC4237445

[69] Ronish L.A., Biswas B., Bauer R.M., Jacob M.E. and Piepenbrink K.H. (2024) The role of extracellular structures in *Clostridioides difficile* biofilm formation. Anaerobe 88, 102873 10.1016/j.anaerobe.2024.102873 38844261

[70] Denis K., Le Bris M., Le Guennec L., Barnier J.P., Faure C., Gouge A. et al. (2019) Targeting Type IV pili as an antivirulence strategy against invasive meningococcal disease. Nat. Microbiol. 4, 972–984 10.1038/s41564-019-0395-8 30911127

[71] Pegden R.S., Larson M.A., Grant R.J. and Morrison M. (1998) Adherence of the Gram-positive bacterium *Ruminococcus albus* to cellulose and identification of a novel form of cellulose-binding protein which belongs to the Pil family of proteins. J. Bacteriol. 180, 5921–5927 10.1128/JB.180.22.5921-5927.1998 9811650 PMC107666

[72] Rakotoarivonina H., Jubelin G., Hebraud M., Gaillard-Martinie B., Forano E. and Mosoni P. (2002) Adhesion to cellulose of the Gram-positive bacterium* Ruminococcus albus* involves type IV pili. Microbiology 148, 1871–1880 10.1099/00221287-148-6-1871 12055307

[73] Vodovnik M., Duncan S.H., Reid M.D., Cantlay L., Turner K., Parkhill J. et al. (2013) Expression of cellulosome components and type IV pili within the extracellular proteome of *Ruminococcus flavefaciens* 007. PloS One 8, e65333 10.1371/journal.pone.0065333 23750253 PMC3672088

[74] Rozman V., Accetto T., Duncan S.H., Flint H.J. and Vodovnik M. (2021) Type IV pili are widespread among non-pathogenic Gram-positive gut bacteria with diverse carbohydrate utilization patterns. Environ. Microbiol. 23, 1527–1540 10.1111/1462-2920.15362 33331146

[75] Craig L., Forest K.T. and Maier B. (2019) Type IV pili: dynamics, biophysics and functional consequences. Nat. Rev. Microbiol. 17, 429–440 10.1038/s41579-019-0195-4 30988511

[76] Wong G.C.L., Antani J.D., Lele P.P., Chen J., Nan B., Kuhn M.J. et al. (2021) Roadmap on emerging concepts in the physical biology of bacterial biofilms: from surface sensing to community formation. Phys. Biol. 18, 5 051501 10.1088/1478-3975/abdc0e 33462162 PMC8506656

[77] Kirkpatrick C.L. and Viollier P.H. (2012) Decoding Caulobacter development. FEMS Microbiol. Rev. 36, 193–205 10.1111/j.1574-6976.2011.00309.x 22091823

[78] Sangermani M., Hug I., Sauter N., Pfohl T. and Jenal U. (2019) Tad pili play a dynamic role in* Caulobacter crescentus* surface colonization. mBio. 10, e01237–19 10.1128/mBio.01237-19 31213565 PMC6581867

[79] Wassmann P., Chan C., Paul R., Beck A., Heerklotz H., Jenal U. et al. (2007) Structure of BeF_3_^-^-modified response regulator PleD: implications for diguanylate cyclase activation, catalysis, and feedback inhibition. Structure 15, 915–927 10.1016/j.str.2007.06.016 17697997

[80] Paul R., Jaeger T., Abel S., Wiederkehr I., Folcher M., Biondi E.G. et al. (2008) Allosteric regulation of histidine kinases by their cognate response regulator determines cell fate. Cell 133, 452–461 10.1016/j.cell.2008.02.045 18455986 PMC2804905

[81] Del Medico L., Cerletti D., Schachle P., Christen M. and Christen B. (2020) The type IV pilin PilA couples surface attachment and cell-cycle initiation in *Caulobacter crescentus*. Proc. Natl. Acad. Sci. U.S.A. 117, 9546–9553 10.1073/pnas.1920143117 32295877 PMC7196804

[82] Ellison C.K., Kan J., Dillard R.S., Kysela D.T., Ducret A., Berne C. et al. (2017) Obstruction of pilus retraction stimulates bacterial surface sensing. Science 358, 535–538 10.1126/science.aan5706 29074778 PMC5805138

[83] Snyder R.A., Ellison C.K., Severin G.B., Whitfield G.B., Waters C.M. and Brun Y.V. (2020) Surface sensing stimulates cellular differentiation in *Caulobacter crescentus*. Proc. Natl. Acad. Sci. U.S.A. 117, 17984–17991 10.1073/pnas.1920291117 32661164 PMC7395532

[84] Hug I., Deshpande S., Sprecher K.S., Pfohl T. and Jenal U. (2017) Second messenger-mediated tactile response by a bacterial rotary motor. Science 358, 531–534 10.1126/science.aan5353 29074777

[85] Obeid Charrouf F., Whitfield G.B., Ellison C.K. and Brun Y.V. (2025) Stimulation of the *Caulobacter crescentus* surface sensing pathway by deletion of a specialized minor pilin-like gene. mBio 16, e0230225 10.1128/mbio.02302-25 41031830 PMC12607857

[86] Gordon V.D. and Wang L. (2019) Bacterial mechanosensing: the force will be with you, always. J. Cell Sci. 132, jcs227694 10.1242/jcs.227694 30944157 PMC6467485

[87] Leighton T.L., Buensuceso R.N., Howell P.L. and Burrows L.L. (2015) Biogenesis of *Pseudomonas aeruginosa* type IV pili and regulation of their function. Environ. Microbiol. 17, 4148–4163 10.1111/1462-2920.12849 25808785

[88] Geiger C.J., Wong G.C.L. and O'Toole G.A. (2024) A bacterial sense of touch: T4P retraction motor as a means of surface sensing by *Pseudomonas aeruginosa* PA14. J. Bacteriol. 206, e0044223 10.1128/jb.00442-23 38832786 PMC11270903

[89] Ellison C.K., Whitfield G.B. and Brun Y.V. (2022) Type IV Pili: dynamic bacterial nanomachines. FEMS Microbiol. Rev. 46, fuab053 10.1093/femsre/fuab053 34788436

[90] Webster S.S., Mathelie-Guinlet M., Verissimo A.F., Schultz D., Viljoen A., Lee C.K. et al. (2021) Force-induced changes of PilY1 drive surface sensing by* Pseudomonas aeruginosa*. mBio 13, e0375421 10.1128/mbio.03754-21 35100866 PMC8806160

[91] Orans J., Johnson M.D., Coggan K.A., Sperlazza J.R., Heiniger R.W., Wolfgang M.C. et al. (2010) Crystal structure analysis reveals *Pseudomonas* PilY1 as an essential calcium-dependent regulator of bacterial surface motility. Proc. Natl. Acad. Sci. U.S.A. 107, 1065–1070 10.1073/pnas.0911616107 20080557 PMC2824316

[92] Schmidt W.C., Lee C.K., Zheng X., Chen J.W., Fetah K.L., Popoli J.R. et al. (2025) *Pseudomonas aeruginosa* senses exopolysaccharide trails using type IV pili and adhesins during biofilm formation. Nat. Microbiol. 10, 2511–2520 10.1038/s41564-025-02087-4 40913087 PMC13267930

[93] Chang C.Y. (2017) Surface sensing for biofilm formation in *Pseudomonas aeruginosa*. Front. Microbiol. 8, 2671 10.3389/fmicb.2017.02671 29375533 PMC5767216

[94] Webster S.S., Lee C.K., Schmidt W.C., Wong G.C.L. and O'Toole G.A. (2021) Interaction between the type 4 pili machinery and a diguanylate cyclase fine-tune c-di-GMP levels during early biofilm formation. Proc. Natl. Acad. Sci. U.S.A. 118, e2105566118 10.1073/pnas.2105566118 34168081 PMC8256011

[95] Maldarelli G.A., Piepenbrink K.H., Scott A.J., Freiberg J.A., Song Y., Achermann Y. et al. (2016) Type IV pili promote early biofilm formation by *Clostridium difficile*. Pathog Dis. 74, ftw061 10.1093/femspd/ftw061 27369898 PMC5985507

[96] Tremblay Y.D.N., Durand B.A.R., Hamiot A., Martin-Verstraete I., Oberkampf M., Monot M. et al. (2021) Metabolic adaption to extracellular pyruvate triggers biofilm formation in *Clostridioides difficile*. ISME J. 15, 3623–3635 10.1038/s41396-021-01042-5 34155333 PMC8630010

[97] Zahavi E.E., Lieberman J.A., Donnenberg M.S., Nitzan M., Baruch K., Rosenshine I. et al. (2011) Bundle-forming pilus retraction enhances enteropathogenic *Escherichia coli* infectivity. Mol. Biol. Cell. 22, 2436–2447 10.1091/mbc.e11-01-0001 21613538 PMC3135470

[98] Cehovin A., Kroll J.S. and Pelicic V. (2011) Testing the vaccine potential of PilV, PilX and ComP, minor subunits of* Neisseria meningitidis* type IV pili. Vaccine 29, 6858–6865 10.1016/j.vaccine.2011.07.060 21803096

[99] Biais N., Ladoux B., Higashi D., So M. and Sheetz M. (2008) Cooperative retraction of bundled type IV pili enables nanonewton force generation. PLoS Biol. 6, e87 10.1371/journal.pbio.0060087 18416602 PMC2292754

[100] Marathe R., Meel C., Schmidt N.C., Dewenter L., Kurre R., Greune L. et al. (2014) Bacterial twitching motility is coordinated by a two-dimensional tug-of-war with directional memory. Nat. Commun. 5, 3759 10.1038/ncomms4759 24806757

[101] Knutton S., Shaw R.K., Anantha R.P., Donnenberg M.S. and Zorgani A.A. (1999) The type IV bundle-forming pilus of enteropathogenic *Escherichia coli* undergoes dramatic alterations in structure associated with bacterial adherence, aggregation and dispersal. Mol. Microbiol. 33, 499–509 10.1046/j.1365-2958.1999.01495.x 10417641

[102] Tobe T. and Sasakawa C. (2001) Role of bundle-forming pilus of enteropathogenic *Escherichia coli* in host cell adherence and in microcolony development. Cell. Microbiol. 3, 579–585 10.1046/j.1462-5822.2001.00136.x 11553010

[103] Saldana Z., Erdem A.L., Schuller S., Okeke I.N., Lucas M., Sivananthan A. et al. (2009) The *Escherichia coli* common pilus and the bundle-forming pilus act in concert during the formation of localized adherence by enteropathogenic *E. coli*. J. Bacteriol. 191, 3451–3461 10.1128/JB.01539-08 19218393 PMC2681888

[104] Kirn T.J., Lafferty M.J., Sandoe C.M. and Taylor R.K. (2000) Delineation of pilin domains required for bacterial association into microcolonies and intestinal colonization by *Vibrio cholerae*. Mol. Microbiol. 35, 896–910 10.1046/j.1365-2958.2000.01764.x 10692166

[105] Helaine S., Dyer D.H., Nassif X., Pelicic V. and Forest K.T. (2007) 3D structure/function analysis of PilX reveals how minor pilins can modulate the virulence properties of type IV pili. Proc. Natl. Acad. Sci. U.S.A. 104, 15888–15893 10.1073/pnas.0707581104 17893339 PMC2000383

[106] Dodson T.A., Carlson E.A., Wamer N.C., Morse C.N., Gadient J.N. and Prestwich E.G. (2022) Characterization of distinct biofilm cell subpopulations and implications in quorum sensing and antibiotic resistance. mBio 13, e0019122 10.1128/mbio.00191-22 35695457 PMC9239111

[107] Sauer K., Stoodley P., Goeres D.M., Hall-Stoodley L., Burmolle M., Stewart P.S. et al. (2022) The biofilm life cycle: expanding the conceptual model of biofilm formation. Nat. Rev. Microbiol. 20, 608–620 10.1038/s41579-022-00767-0 35922483 PMC9841534

[108] Drescher K., Shen Y., Bassler B.L. and Stone H.A. (2013) Biofilm streamers cause catastrophic disruption of flow with consequences for environmental and medical systems. Proc. Natl. Acad. Sci. U.S.A. 110, 4345–4350 10.1073/pnas.1300321110 23401501 PMC3600445

[109] Jude B.A. and Taylor R.K. (2011) The physical basis of type 4 pilus-mediated microcolony formation by *Vibrio cholerae* O1. J. Struct. Biol. 175, 1–9 10.1016/j.jsb.2011.04.008 21527347 PMC3102138

[110] Reguera G. and Kolter R. (2005) Virulence and the environment: a novel role for* Vibrio cholerae* toxin-coregulated pili in biofilm formation on chitin. J. Bacteriol. 187, 3551–3555 10.1128/JB.187.10.3551-3555.2005 15866944 PMC1112007

[111] Liu H.H., Yang Y.R., Shen X.C., Zhang Z.L., Shen P. and Xie Z.X. (2008) Role of DNA in bacterial aggregation. Curr. Microbiol. 57, 139–144 10.1007/s00284-008-9166-0 18491189

[112] Das T., Sharma P.K., Busscher H.J., van der Mei H.C. and Krom B.P. (2010) Role of extracellular DNA in initial bacterial adhesion and surface aggregation. Appl. Environ. Microbiol. 76, 3405–3408 10.1128/AEM.03119-09 20363802 PMC2869138

[113] Das T., Krom B.P., van der Mei H.C., Busscher H.J. and Sharma P.K. (2011) DNA-mediated bacterial aggregation is dictated by acid-base interactions. Soft Matter 7, 2927–2935 10.1039/c0sm01142h

[114] Kong Y., Du Q., Zhao D., Wen Y., Zhang T., Geng Z. et al. (2025) DNA-programmed responsive microorganism assembly with controlled patterns and behaviors. Sci. Adv. 11, eads8651 10.1126/sciadv.ads8651 40512851 PMC12164988

[115] Neil R.B. and Apicella M.A. (2009) Clinical and laboratory evidence for *Neisseria meningitidis* biofilms. Future Microbiol. 4, 555–563 10.2217/fmb.09.27 19492966 PMC2827931

[116] McCarthy R.R. (2022) Targeting bacterial virulence to tackle the antimicrobial resistance crisis. In GARDP Antimicrobial Viewpoints(Piddock L., Pentz-Murr A., Santu A., Kouassi V. and Derakhshani S., eds), pp. 1–5, Global Antibiotic Research & Development Partnership (GARDP), Geneva 41489896

[117] Clatworthy A.E., Pierson E. and Hung D.T. (2007) Targeting virulence: a new paradigm for antimicrobial therapy. Nat. Chem. Biol. 3, 541–548 10.1038/nchembio.2007.24 17710100

[118] Allen R.C., Popat R., Diggle S.P. and Brown S.P. (2014) Targeting virulence: can we make evolution-proof drugs? Nat. Rev. Microbiol. 12, 300–308 10.1038/nrmicro3232 24625893

[119] Fleitas Martinez O., Cardoso M.H., Ribeiro S.M. and Franco O.L. (2019) Recent advances in anti-virulence therapeutic strategies with a focus on dismantling bacterial membrane microdomains, toxin neutralization, quorum-sensing interference and biofilm inhibition. Front. Cell Infect. Microbiol. 9, 74 10.3389/fcimb.2019.00074 31001485 PMC6454102

[120] Dieltjens L., Appermans K., Lissens M., Lories B., Kim W., Van der Eycken E.V. et al. (2020) Inhibiting bacterial cooperation is an evolutionarily robust anti-biofilm strategy. Nat. Commun. 11, 107 10.1038/s41467-019-13660-x 31919364 PMC6952394

[121] Gabaldon T. (2023) Nothing makes sense in drug resistance except in the light of evolution. Curr. Opin. Microbiol. 75, 102350 10.1016/j.mib.2023.102350 37348192

[122] Solanki V., Kapoor S. and Thakur K.G. (2018) Structural insights into the mechanism of Type IVa pilus extension and retraction ATPase motors. FEBS J. 285, 3402–3421 10.1111/febs.14619 30066435

[123] Shimozono T.M., Vogelaar N.J., O'Hara M.T. and Yang Z. (2025) A phage-based approach to identify antivirulence inhibitors of bacterial type IV Pili. Microb. Biotechnol. 18, e70081 10.1111/1751-7915.70081 39822166 PMC11739798

[124] Ellison C.K., Dalia T.N., Dalia A.B. and Brun Y.V. (2019) Real-time microscopy and physical perturbation of bacterial pili using maleimide-conjugated molecules. Nat. Protoc. 14, 1803–1819 10.1038/s41596-019-0162-6 31028374 PMC7461830

[125] Amaral L., Kristiansen J.E., Viveiros M. and Atouguia J. (2001) Activity of phenothiazines against antibiotic-resistant *Mycobacterium tuberculosis*: a review supporting further studies that may elucidate the potential use of thioridazine as anti-tuberculosis therapy. J. Antimicrob. Chemother. 47, 505–511 10.1093/jac/47.5.505 11328759

[126] Kristiansen M.M., Leandro C., Ordway D., Martins M., Viveiros M., Pacheco T. et al. (2003) Phenothiazines alter resistance of methicillin-resistant strains of *Staphylococcus aureus* (MRSA) to oxacillin* in vitro*. Int. J. Antimicrob. Agents 22, 250–253 10.1016/S0924-8579(03)00200-0 13678829

[127] Thanacoody H.K. (2007) Thioridazine: resurrection as an antimicrobial agent? Br. J. Clin. Pharmacol. 64, 566–574 10.1111/j.1365-2125.2007.03021.x 17764469 PMC2203271

[128] Ohlow M.J. and Moosmann B. (2011) Phenothiazine: the seven lives of pharmacology’s first lead structure. Drug Discov. Today 16, 119–131 10.1016/j.drudis.2011.01.001 21237283

[129] Thiele R. (1965) Triflupromazine as a tranquilizer and antiemetic drug in internal medicine. Med. Klin. 60, 1503–1504 5862106

[130] Leucht S., Cipriani A., Spineli L., Mavridis D., Orey D., Richter F. et al. (2013) Comparative efficacy and tolerability of 15 antipsychotic drugs in schizophrenia: a multiple-treatments meta-analysis. Lancet 382, 951–962 10.1016/S0140-6736(13)60733-3 23810019

[131] Dye K.J., Vogelaar N.J., O'Hara M., Sobrado P., Santos W., Carlier P.R. et al. (2022) Discovery of two inhibitors of the type IV pilus assembly ATPase PilB as potential antivirulence compounds. Microbiol. Spectr. 10, e0387722 10.1128/spectrum.03877-22 36377931 PMC9769694

[132] McDonald-Ramos J.S., Hicklin I.K., Yang Z. and Brown A.M. (2024) Identification of small molecule inhibitors of the *Chloracidobacterium thermophilum* type IV pilus protein PilB by ensemble virtual screening. Arch. Biochem. Biophys. 760, 110127 10.1016/j.abb.2024.110127 39154818

[133] Dumenil G. (2019) Type IV pili as a therapeutic target. Trends Microbiol. 27, 658–661 10.1016/j.tim.2019.05.005 31182345

[134] Maldarelli G.A., De Masi L., von Rosenvinge E.C., Carter M. and Donnenberg M.S. (2014) Identification, immunogenicity, and cross-reactivity of type IV pilin and pilin-like proteins from *Clostridium difficile*. Pathog Dis. 71, 302–314 10.1111/2049-632X.12137 24550179 PMC4130776

[135] Charlebois A., Deslauriers N., Maduro L. and Boulianne M. (2025) Immunogenicity of type IV pilin proteins from *Clostridium perfringens* in chickens. Microorganisms 13, 120 10.3390/microorganisms13010120 39858888 PMC11767711

[136] Fernandez-Martinez D., Kong Y., Goussard S., Zavala A., Gastineau P., Rey M. et al. (2024) Cryo-EM structures of type IV pili complexed with nanobodies reveal immune escape mechanisms. Nat. Commun. 15, 2414 10.1038/s41467-024-46677-y 38499587 PMC10948894

[137] Li Y., Lux R., Pelling A.E., Gimzewski J.K. and Shi W. (2005) Analysis of type IV pilus and its associated motility in *Myxococcus xanthus* using an antibody reactive with native pilin and pili. Microbiology (Reading) 151, 353–360 10.1099/mic.0.27614-0 15699186

[138] Novotny L.A., Jurcisek J.A., Ward M.O. Jr, Jordan Z.B., Goodman S.D. and Bakaletz L.O. (2015) Antibodies against the majority subunit of type IV Pili disperse nontypeable *Haemophilus influenzae* biofilms in a LuxS-dependent manner and confer therapeutic resolution of experimental otitis media. Mol. Microbiol. 96, 276–292 10.1111/mmi.12934 25597921 PMC4423401

[139] Mokrzan E.M., Novotny L.A., Brockman K.L. and Bakaletz L.O. (2018) Antibodies against the majority subunit (PilA) of the type IV pilus of nontypeable *Haemophilus influenzae* disperse *Moraxella catarrhalis* from a dual-species biofilm. mBio. 9, e02423–18 10.1128/mBio.02423-18 30538189 PMC6299487

[140] Jurcisek J.A., Hofer L.K., Goodman S.D. and Bakaletz L.O. (2022) Monoclonal antibodies that target extracellular DNABII proteins or the type IV pilus of nontypeable Haemophilus influenzae (NTHI) worked additively to disrupt 2-genera biofilms. Biofilm 4, 100096 10.1016/j.bioflm.2022.100096 36532267 PMC9747592

[141] Abu Resha R.A. and Al-Zubaidy B.B. (2023) Study the prophylactic role of anti-type IV pili (fimbriae) antibody against pulmonary infection caused by *P. aeruginosa in vivo* (mice). Iraqi J. Sci. 56, 998–1008

[142] Egerton J.R., Cox P.T., Anderson B.J., Kristo C., Norman M. and Mattick J.S. (1987) Protection of sheep against footrot with a recombinant DNA-based fimbrial vaccine. Vet. Microbiol. 14, 393–409 10.1016/0378-1135(87)90030-7 2891214

[143] Hunt J.D., Jackson D.C., Brown L.E., Wood P.R. and Stewart D.J. (1994) Antigenic competition in a multivalent foot rot vaccine. Vaccine 12, 457–464 10.1016/0264-410X(94)90125-2 7912871

[144] McPherson A.S., Dhungyel O.P. and Whittington R.J. (2018) Detection and serogrouping of *Dichelobacter nodosus* infection by use of direct PCR from lesion swabs to support outbreak-specific vaccination for virulent footrot in sheep. J. Clin. Microbiol. 56, e01730–17 10.1128/JCM.01730-17 29436426 PMC5869834

[145] Kao D.J., Churchill M.E., Irvin R.T. and Hodges R.S. (2007) Animal protection and structural studies of a consensus sequence vaccine targeting the receptor binding domain of the type IV pilus of *Pseudomonas aeruginosa*. J. Mol. Biol. 374, 426–442 10.1016/j.jmb.2007.09.032 17936788 PMC3493149

[146] Kao D.J. and Hodges R.S. (2009) Advantages of a synthetic peptide immunogen over a protein immunogen in the development of an anti-pilus vaccine for *Pseudomonas aeruginosa*. Chem. Biol. Drug Des. 74, 33–42 10.1111/j.1747-0285.2009.00825.x 19519742 PMC2756486

[147] Rollenhagen J.E., Kalsy A., Cerda F., John M., Harris J.B., Larocque R.C. et al. (2006) Transcutaneous immunization with toxin-coregulated pilin A induces protective immunity against *Vibrio cholerae* O1 El Tor challenge in mice. Infect. Immun. 74, 5834–5839 10.1128/IAI.00438-06 16988262 PMC1594919

[148] Horzempa J., Held T.K., Cross A.S., Furst D., Qutyan M., Neely A.N. et al. (2008) Immunization with a *Pseudomonas aeruginosa* 1244 pilin provides O-antigen-specific protection. Clin. Vaccine Immunol. 15, 590–597 10.1128/CVI.00476-07 18272666 PMC2292668

[149] Arefian Jazi M., Hajikhani B., Goudarzi M. and Ebrahimipour G. (2024) Exploiting immunopotential PAPI-1 encoded type IVb major pilin targeting *Pseudomonas aeruginosa*. Heliyon 10, e36859 10.1016/j.heliyon.2024.e36859 39281519 PMC11401190

[150] Maldarelli G.A., Matz H., Gao S., Chen K., Hamza T., Yfantis H.G. et al. (2016) Pilin vaccination stimulates weak antibody responses and provides no protection in a C57Bl/6 murine model of acute *Clostridium difficile* infection. J. Vaccines Vaccin. 7, 321 10.4172/2157-7560.1000321 27375958 PMC4927082

[151] Fernandes P.J., Guo Q., Waag D.M. and Donnenberg M.S. (2007) The type IV pilin of *Burkholderia mallei* is highly immunogenic but fails to protect against lethal aerosol challenge in a murine model. Infect. Immun. 75, 3027–3032 10.1128/IAI.00150-07 17403869 PMC1932848

[152] Lu Y.C., Li M.C., Chen Y.M., Chu C.Y., Lin S.F. and Yang W.J. (2011) DNA vaccine encoding type IV pilin of *Actinobacillus pleuropneumoniae* induces strong immune response but confers limited protective efficacy against serotype 2 challenge. Vaccine 29, 7740–7746 10.1016/j.vaccine.2011.07.127 21835218

[153] Harding C.M., Nasr M.A., Kinsella R.L., Scott N.E., Foster L.J., Weber B.S. et al. (2015) Acinetobacter strains carry two functional oligosaccharyltransferases, one devoted exclusively to type IV pilin, and the other one dedicated to O-glycosylation of multiple proteins. Mol. Microbiol. 96, 1023–1041 10.1111/mmi.12986 25727908

[154] Borud B. and Koomey M. (2024) Sweet complexity: O-linked protein glycosylation in pathogenic Neisseria. Front. Cell Infect. Microbiol. 14, 1407863 10.3389/fcimb.2024.1407863 38808060 PMC11130364

[155] Meng E.C., Goddard T.D., Pettersen E.F., Couch G.S., Pearson Z.J., Morris J.H. et al. (2023) UCSF ChimeraX: tools for structure building and analysis. Protein Sci. 32, e4792 10.1002/pro.4792 37774136 PMC10588335

[156] Jumper J., Evans R., Pritzel A., Green T., Figurnov M., Ronneberger O. et al. (2021) Highly accurate protein structure prediction with AlphaFold. Nature 596, 583–589 10.1038/s41586-021-03819-2 34265844 PMC8371605

[157] Sievers F., Wilm A., Dineen D., Gibson T.J., Karplus K., Li W. et al. (2011) Fast, scalable generation of high-quality protein multiple sequence alignments using Clustal Omega. Mol. Syst. Biol. 7, 539 10.1038/msb.2011.75 21988835 PMC3261699

[158] Letunic I. and Bork P. (2024) Interactive Tree of Life (iTOL) v6: recent updates to the phylogenetic tree display and annotation tool. Nucleic Acids Res. 52, W78–W82 10.1093/nar/gkae268 38613393 PMC11223838

[159] Thongchol J., Yu Z., Harb L., Lin Y., Koch M., Theodore M. et al. (2024) Removal of *Pseudomonas* type IV pili by a small RNA virus. Science 384, eadl0635 10.1126/science.adl0635 38574145 PMC11126211

